# The role of mathematical modelling in understanding prokaryotic predation

**DOI:** 10.3389/fmicb.2022.1037407

**Published:** 2022-12-29

**Authors:** J. Kimberley Summers, Jan-Ulrich Kreft

**Affiliations:** ^1^Wellington Lab, School of Life Sciences, University of Warwick, Coventry, United Kingdom; ^2^Kreft Lab, Institute of Microbiology and Infection and Centre for Computational Biology and School of Biosciences, University of Birmingham, Edgbaston, Birmingham, United Kingdom

**Keywords:** predator–prey models, *Bdellovibrio bacteriovorus*, ecological models, AMR, optimal foraging theory, ecosystems, robust permanence

## Abstract

With increasing levels of antimicrobial resistance impacting both human and animal health, novel means of treating resistant infections are urgently needed. Bacteriophages and predatory bacteria such as *Bdellovibrio bacteriovorus* have been proposed as suitable candidates for this role. Microbes also play a key environmental role as producers or recyclers of nutrients such as carbon and nitrogen, and predators have the capacity to be keystone species within microbial communities. To date, many studies have looked at the mechanisms of action of prokaryotic predators, their safety in *in vivo* models and their role and effectiveness under specific conditions. Mathematical models however allow researchers to investigate a wider range of scenarios, including aspects of predation that would be difficult, expensive, or time-consuming to investigate experimentally. We review here a history of modelling in prokaryote predation, from simple Lotka-Volterra models, through increasing levels of complexity, including multiple prey and predator species, and environmental and spatial factors. We consider how models have helped address questions around the mechanisms of action of predators and have allowed researchers to make predictions of the dynamics of predator–prey systems. We examine what models can tell us about qualitative and quantitative commonalities or differences between bacterial predators and bacteriophage or protists. We also highlight how models can address real-world situations such as the likely effectiveness of predators in removing prey species and their potential effects in shaping ecosystems. Finally, we look at research questions that are still to be addressed where models could be of benefit.

## Introduction

*“Tyger tyger, burning bright,*

*In the forests of the night;*

*What immortal hand or eye,*

*Could frame thy fearful symmetry?”*

With his evocative poem, Blake (1794) illustrates the sense of fascination we have for predators, from the tiger in the poem, to a wolf pack or a spider waiting in her web. We feel a sense of excitement in the hunt and anthropomorphically identify with both the hunter and the hunted. At the microscopic level, bacteria are under attack from a wide range of predators from rotifers, nematodes like *Caenorhabditis elegans*, protists such as amoebae, flagellates, and ciliates, to bacteriophages and even other bacteria, such as *Bdellovibrio bacteriovorus*. For more details on predators of bacteria see the review by Hungate et al. (2021). Despite this, microbial predators rarely evoke the same sense of awe, yet they share many properties of the relationships of larger predators and prey, and can make excellent models, as well as being fascinating and important in their own right. Predation of bacteria by protists (Curds, 1973; Habte and Alexander, 1978b; Fuhrman and Noble, 1995; Jürgens and Matz, 2002; Pernthaler, 2005), bacteriophages (Proctor and Fuhrman, 1990) and other bacteria (Williams et al., 2016), plays an important ecological role by regulating the density of bacteria, which are producers and recyclers of carbon and nitrogen (Azam et al., 1983). In medicine, animal and plant agriculture and aquaculture, with the increase in antimicrobial resistance, new safe and effective treatments are constantly being sought to treat bacterial infections, and predators, such as bacteriophage (Sulakvelidze et al., 2001; Atterbury et al., 2007; Struelens et al., 2009; Abedon et al., 2011; Golkar et al., 2014) and *B. bacteriovorus* (Scherff, 1973; Chu and Zhu, 2009; Atterbury et al., 2011; Willis et al., 2016; Baker et al., 2017; Shatzkes et al., 2017) have been proposed for this purpose.

The use of predatory bacteria, such as *B. bacteriovorus*, is an attractive option for treating drug resistant infections, as they predate a wide range of Gram-negative bacteria regardless of any drug resistance (Kadouri, 2011; Sun et al., 2017; Dharani et al., 2018; Jang et al., 2021). Several studies have addressed the issue of safety of *Bdellovibrio* and like organisms (BALOs; Lenz and Hespell, 1978; Shatzkes et al., 2015; Gupta et al., 2016; Monnappa et al., 2016) and found BALOs to be non-toxic to eukaryotic cells and only mildly immunogenic (Gupta et al., 2016; Shatzkes et al., 2016, 2017), probably due to their unusual lipid A (Schwudke et al., 2003). The effectiveness of BALOs in treating various bacterial infections has been investigated in plants (Scherff, 1973; Uematsu, 1980; Paulis, 2017; Youdkes et al., 2020), corals (Welsh et al., 2017), fungi (Saxon et al., 2014), nematodes (Emmert et al., 2014), fruit flies (Sivakala et al., 2021), mice, rats and rabbits (Shatzkes et al., 2016, 2017; Russo et al., 2018; Findlay et al., 2019; Sar et al., 2020; Romanowski et al., 2021), farm animals (Atterbury et al., 2011; Boileau et al., 2011, 2016) and aquaculture (Chu and Zhu, 2009; Richards et al., 2012; Li et al., 2014; Cao et al., 2015, 2020; Guo et al., 2017; Ottaviani et al., 2020; Ooi et al., 2021), as well as in combination with the immune system (Willis et al., 2016) or antibiotics (Im et al., 2017a). Meanwhile, a great many studies have explored the possibilities of treating infections with bacteriophages over the last 100 years (Gordillo Altamirano and Barr, 2019; Hatfull et al., 2022).

Which of BALOs or bacteriophage is the better option for treating a resistant infection is likely to be dependent on multiple factors – see Table 1. In general, *B. bacteriovorus* is a better choice when a broad-spectrum approach is required or acquired resistance is considered an issue, as well as in spatially complex environments, such as biofilms. Bacteriophage are more appropriate when a targeted approached is needed, when speed is of the essence or in anoxic environments (as all known BALOs are aerobes). Regardless of which predator is most suitable in any particular situation, we need to better understand the dynamics of microbial predator–prey interactions to make best use of it and mathematical modelling can be an invaluable tool to do so.

**Table 1:** Comparison of the properties of BALOs and bacteriophage regarding treating bacterial infections.

Predator	BALO	Bacteriophage	References
Specialist or generalist	Generalist	Specialist	(Cohen et al., 2021)
Motile	Yes	No	(Lambert et al., 2006)
Has chemotaxis	Yes	No	(Lambert et al., 2003)
Can predate dormant cells	Yes	No	(Markelova, 2010)
Can move through biofilms	Yes	Only by diffusion	(Núñez et al., 2005)
Resistance	Phenotypic is common, genetically inherited is rare	Develops rapidly	(Luria and Delbrück, 1943; Shemesh and Jurkevitch, 2004)
Requires oxygen	Yes	No	(Stolp and Starr, 1963)
Lysis time	3–4 h	23–36 min	(Shilo and Bruff, 1965; Hadas et al., 1997; De Paepe and Taddei, 2006)
Burst size (from E. coli prey)	3–6	50–150	(Seidler and Starr, 1969b; Hadas et al., 1997; De Paepe and Taddei, 2006)

There is a range of lysis times and burst sizes, the numbers given are typical for T phages.

## The role of mathematical modelling

The role of mathematical modelling in microbial predation is three-fold. Firstly, in collaboration with experimental work, to assist in understanding the mechanisms and kinetics of predation, such as how and why resistance to predation occurs and reverts, given the trade-off between its obvious benefits and potential costs, and to explore the role of chemotaxis in optimizing *B. bacteriovorus’s* hunting capabilities. Secondly, to gain an understanding of when predators are likely to eliminate (or very substantially reduce the numbers of) prey and when they are likely to stabilize a more complex microbial community consisting of multiple competing and co-operating species. The former is of particular importance when designing treatments for infections, whilst both elimination and stabilization can be important in ecological studies. Finally mathematical modelling can help support or discount ecological theories of predation such as “kill the winner” or optimal foraging theory and can assist in the design of laboratory studies to test these theories. Whilst all three roles of modelling can play an important part in understanding both ecology and the potential effectiveness of interventions, the number of studies fulfilling these roles vary greatly.

In particular, there is a need to partner mathematical models with experimental data or field studies. By testing models against laboratory data, it is possible to not only reject less suitable models and ensure we are not misled by these, but also to fulfil the above role of mechanistic understanding and to gain improved parameter estimates to inform future models. This in turn can lead to improved experimental design, in which for example predators and prey can be combined in ways which are predicted by the models to lead to certain outcomes, such as the elimination of the prey or the stabilization of a complex community to confirm or refute these predictions. Finally, by multiple iterations of model refinement and experimental validation we can hope to produce models than can capture and predict emergent behavior of complex multi-species systems, including the effects of spatial distribution and species diversity, and explore “what if” scenarios that would be costly, time-consuming, or otherwise impractical to investigate in laboratory or field conditions. Models can also be validated by observations from natural environments such as the abundance and distribution of *B. bacteriovorus* in different environments (Summers and Kreft, 2021) in an approach known as pattern-oriented modelling (Grimm et al., 2005).

## Brief biology relevant to modelling of predator prey dynamics

*B. bacteriovorus* is a small, Gram-negative bacterium around one seventh the volume of *Escherichia coli* (Cover et al., 1984). This means that unlike most macro-predators and indeed protists, but like bacteriophage, *B. bacteriovorus* can gain enough resources from a single prey item to produce multiple new predators, which is in fact necessary for survival (Summers and Kreft, 2021). Given that *B. bacteriovorus* enters inside its prey prior to consuming it and only predates once in its life cycle, commentators have questioned whether it should be considered a true predator. Indeed, early works on *B. bacteriovorus* often referred to it as a parasite (Seidler and Starr, 1968; Varon and Shilo, 1969; Burnham et al., 1970). The term parasite however fails to capture the nature of the lifestyle of *B. bacteriovorus* given that parasites, whilst impacting fitness, do not generally kill their hosts, something *B. bacteriovorus* undoubtably does. Perhaps the term parasitoid, which are organisms that kill their hosts, may be closer to the truth, yet even here there are differences. Parasitoids often live and grow within their hosts for considerable time before killing them, while *B. bacteriovorus* kills its host / prey almost immediately upon entry into the periplasm. Additionally, parasitoids are generally density independent attackers, that is multiple parasitoids may grow within the same host, whilst *B. bacteriovorus* is very much a solitary attacker. In truth, there is probably no existing definition which captures all the unique aspects of *B. bacteriovorus* predation. Hence, given that parasitoids and true predators generally have a similar impact on the population dynamics of their hosts/prey, and following common usage, we have chosen to use the term predator to describe the behavior of *B. bacteriovorus* within this review.

Wild type *B. bacteriovorus* can predate a wide range of other Gram-negative bacteria (Stolp and Starr, 1963; although the exact prey range varies greatly between strains) and is thus considered a generalist predator similarly to many protists, unlike bacteriophage which are usually specialists (Cohen et al., 2021), although this may in part be biased by the usual isolation on single hosts (Yu et al., 2017). It has a bi-phasic lifestyle, consisting of an attack phase and a growth phase. During its attack phase, *B. bacteriovorus* can swim at speeds of up to 160 μm s^−1^ (roughly 100 body lengths per second; Lambert et al., 2006). Such fast swimming increases its rate of encounter with other bacteria, particularly compared to bacteriophage, which simply drift around, but comes at a cost, as it is responsible for its high endogenous respiration rate, and short half-life (about 10 h) outside of its prey (Hespell et al., 1974). *B. bacteriovorus*’s search for prey is likely improved by chemotaxis, not to detect individual prey but to detect areas of high bacterial density such as biofilms (Garst et al., 2013; Hughes, 2014). The *B. bacteriovorus* genome contains genes involved in chemotaxis and knocking these out can reduce predation efficiency (Lambert et al., 2003). It has also been shown that *B. bacteriovorus* is chemotactically attracted to yeast extracts (Straley et al., 1974), casamino acids (Chauhan and Williams, 2006), high densities of prey cells (Straley and Conti, 1977), and various other compounds, including certain amino acids (Lamarre et al., 1977), short chain fatty acids, components of the tricarboxylic acid cycle and other key metabolic pathways, inorganic ions and oxygen (Straley et al., 1979).

When *B. bacteriovorus* locates a potential prey bacterium, it initially attaches loosely to it (Starr and Baigent, 1966). Attachment to a non-suitable target is reversed after a few minutes (Hobley et al., 2006). With suitable prey, attachment becomes firm and is followed by penetration into the prey periplasm and loss of the flagellum (Starr and Baigent, 1966), a process that takes about 20 min (Varon and Shilo, 1968). During this time, *B. bacteriovorus* kills the prey cell by causing its inner membrane to become porous (Romo et al., 1992), a process which also allows nutrients to leak out into the periplasm. Once inside the periplasm, the predator causes further alterations to the prey peptidoglycan, preventing further degradation (Thomashow and Rittenberg, 1978) and closing the entrance pore in the outer membrane to trap the prey nutrients in the periplasmic space, where the growing predator can absorb them. This growth phase lasts for approximately 3 h (Shilo and Bruff, 1965), a relatively long time considering the predator’s short half-life outside of prey. Whilst this once in a life-cycle event is taking place, the predator is locked away and does not predate any more prey. In this regard, *B. bacteriovorus* is unlike other predators, such as filter feeding protists or most macro-predators. Indeed, the closest comparison might be to a snake that feeds at large intervals and can take a week to digest their food. Unlike a snake, *B. bacteriovorus* feeds only once in its lifespan, making it in some ways closer to a bacteriophage, however in the case of the phage the occlusion period is much shorter, especially relative to its half-life outside of prey. The long “digestion” time of *B. bacteriovorus* means it has a long handling time for prey, the time it takes for a predator from catching to eating and digesting the prey. A predator’s handling time leads to saturation of its rate of prey consumption with increasing prey density (its functional response) and affects its optimal strategy for foraging and selecting prey (Jeschke et al., 2002; Kisdi and Liu, 2006).

Unlike macro-predators and filter feeding protists, *B. bacteriovorus* does not possess the capacity to assess the value of its potential prey [although it can tell a Gram-negative from a Gram-positive bacterium and also identify and reject prey that contains another *B. bacteriovorus* cell (Lerner et al., 2012)]. Additionally, it does not have the opportunity to adapt its behavior from past experience, as predation is a once in a life-time event and as a bacterium, memory and cognition are very limited. The lack of ability to evaluate prey value is somewhat negated by the fact that during its growth phase, *B. bacteriovorus* grows into a long, coenocytic filament (Lambert et al., 2006), until all the prey resources have been absorbed. The filament then septates to give new predators [typically between 3 and 6 from *E. coli* prey (Seidler and Starr, 1969a)], and the prey cell is lysed to release the new *B. bacteriovorus* to search for fresh prey. This unusual form of bacterial growth means *B. bacteriovorus* can form as many new cells as it has the resources for, unlike bacteria which divide by binary fission which can only produce numbers of off-spring that are powers of 2, e.g., 4 or 8 but not 5, 6 or 7, which would often waste resources. In cases of particularly high multiplicity of infection, it is possible for two *B. bacteriovorus* to attach to and penetrate the same prey simultaneously. Tailgating “infections”, where one *B. bacteriovorus* has already established itself in a prey bacterium, and a second *B. bacteriovorus* then penetrates the same prey, are rarer (Lerner et al., 2012).

Whilst *B. bacteriovorus* is one of the most studied bacterial predators, it is far from the only one. A detailed description of other bacterial predators is outside of the scope of this review, interested readers should consult previous reviews of bacterial predation (Jurkevitch and Davidov, 2006; Pérez et al., 2016; Jurkevitch and Mitchell, 2020). In general, bacterial predators range from obligate predators to facultative hunters, and those that excrete toxins, probably more as a way of removing competition than as a source of nutrients. Some bacterial predators, such as *B. bacteriovorus* are lone hunters, whilst others like myxobacteria, e.g., *Myxococcus xanthus*, attack as a group using the so called “wolf-pack” technique (Marshall and Whitworth, 2019), still others use sticky filaments to catch bacteria (Lewin, 1997), in a process more reminiscent of many protist predators. The difference between lone predators and group hunters is an important one, as with the social predators, a critical mass of predators is required for successful predation. This means that at low predator density, predation is inefficient and indeed it is notable that *M. xanthus*, as well as being a group hunter, is also a facultative predator. As such, at a low prey or predator densities, it can reproduce on environmental nutrients, turning to predation only when there are high densities of both prey and predator bacteria (Jurkevitch and Davidov, 2006).

## The original predator prey model of Lotka and Volterra

Whilst there is great potential for the use of mathematical modelling in prokaryotic predation there has been limited use of it to date, despite the rich history over the last century of mathematical modelling of predator–prey dynamics. Indeed most models of microbial predator prey interactions have been tailored to protist predation of bacteria, or bacteriophage infection (Curds and Bazin, 1977; Levin et al., 1977; Bohannan and Lenski, 2000; Krysiak-Baltyn et al., 2016).

One of the earliest predator–prey models (Eqs. 1a and 1b), was developed independently by Lotka (1925) and Volterra (1926), the latter inspired by catches of fish in the Adriatic Sea.


(1a) $$\frac{dN}{dt}=\mu N-\alpha NP$$

(1b) $$\frac{dP}{dt}=\beta NP-\lambda P$$

Where N is the prey and P the predator population size. The prey Eq. (1a) has a term for exponential growth with specific growth rate *μ* followed by a term for prey removal by predation, with a rate proportional to the probability of prey and predator encountering each other (which is proportional to both prey and predator densities, thus proportional to the product of their densities). The predator Eq. (1b) has the same predation term but now with a positive sign and a different ‘conversion factor’, these constants α and β reflect the amount of prey consumed to produce a certain number of predators. The second term describes the mortality of the predator with rate λ.

**Figure 1:**
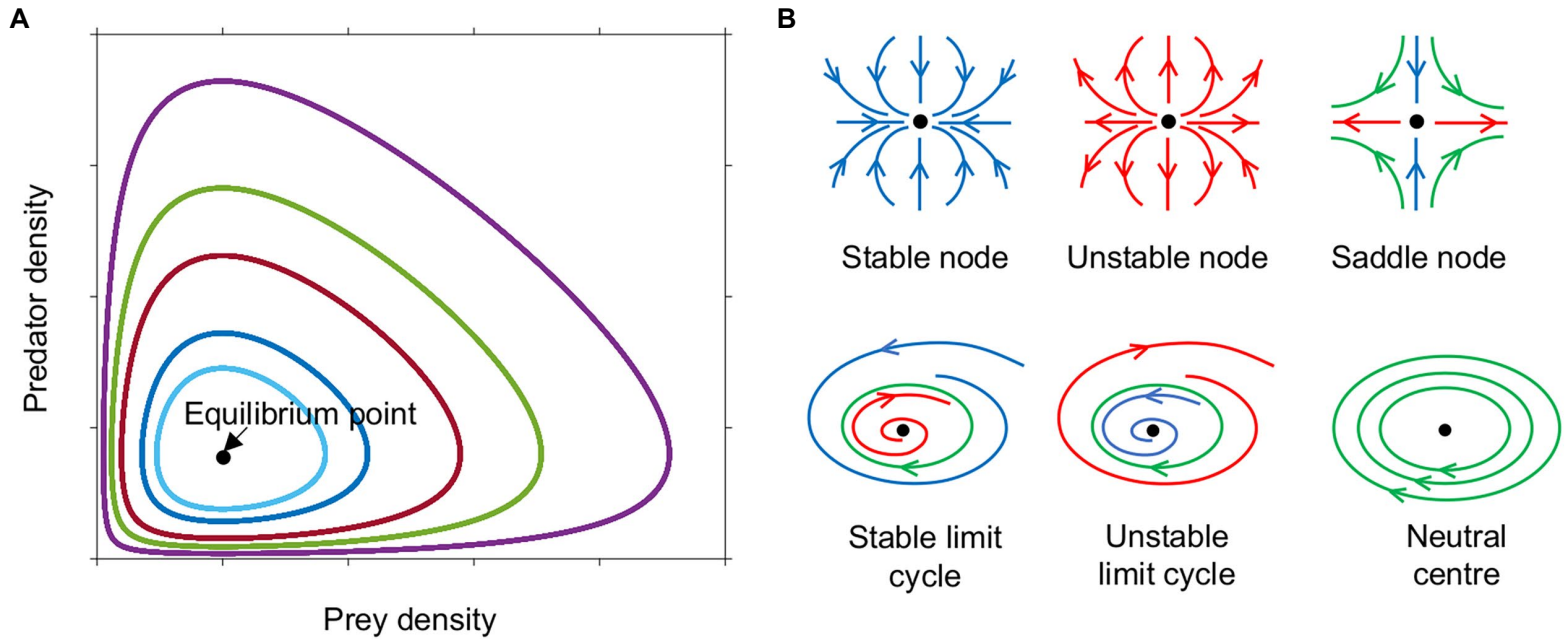
Phase plane diagram of ‘equilibrium’ points or dynamic steady states. **(A)** Phase plane diagram of a Lotka-Volterra system with different initial conditions of prey and predator density indicated by colour. **(B)** Phase plane trajectories of various types of equilibria. Arrows indicate direction of flow over time, blue towards the equilibrium point (black dot), red away from equilibrium, green around the equilibrium. Neutral centers occur in the Lotka-Volterra model (Figure 2A). When this model is modified to have logistic prey growth and a Holling type II predator functional response (Figure 2B), saddle nodes and stable limit cycles are observed. Monod kinetics for the prey combined with a Holling type II predator response (Figures 2C–F) can result in stable nodes, saddle nodes and stable limit cycles.

**Figure 2:**
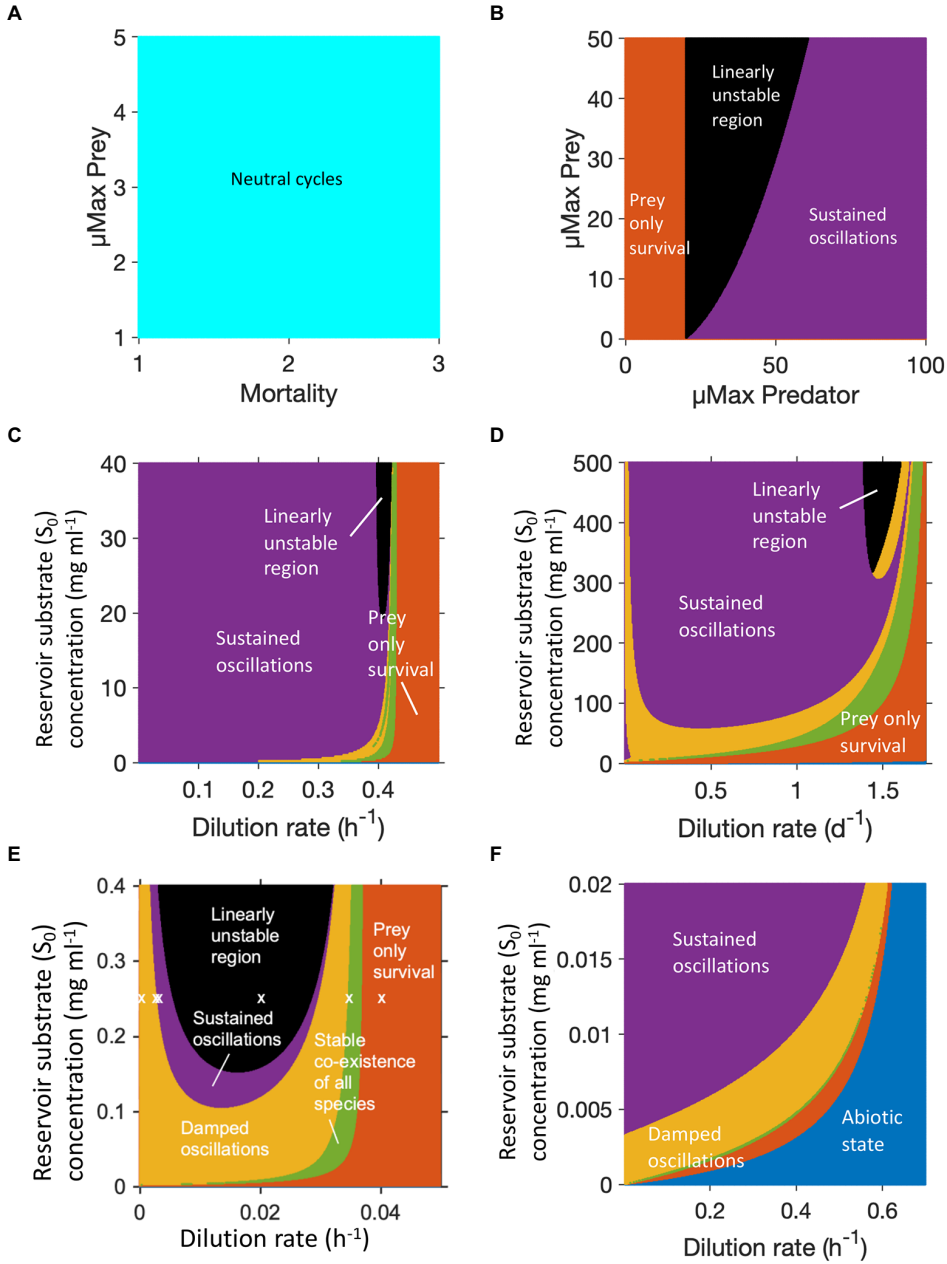
Dynamic regimes generated by different predator prey models, each color represents a different regime. **(A)** Lotka-Volterra model: only neutral cycles. **(B)** Lotka-Volterra model altered to have logistic prey growth and a Holling type II predator functional response: stable limit cycles (sustained oscillations), no coexistence without oscillations. **(C)** Protist predation from (Curds and Bazin, 1977): damped oscillations and coexistence without oscillations possible. **(D)** Rotifer predation from (Fussmann et al., 2000): similar to **(C)** with larger region of damped oscillations. **(E)** *B. bacteriovorus* predation from (Summers and Kreft, 2021). Copyright © 2022 American Society for Microbiology. *Appl. Environ. Microbiol.* 88:e01082-21. doi: 10.1128/AEM.01082-21: similar to **(C)** with even wider region of damped oscillations but also wider linearly unstable region. **(F)** Bacteriophage predation from (Summers and Kreft, 2021): same model/equations as in **(E)** but applying phage parameters removes the linearly unstable region. Note we cannot cover the whole feasible parameter space in these figures.

Lotka and Volterra showed that these interactions could result in oscillatory behavior without the need for any external fluctuations. The oscillations in their model, however, were not stable, as they were cycles about a neutral center, the equilibrium point at $N=\frac{\lambda }{\beta },P=\frac{\mu }{\alpha },$ that was neither an attractor, nor a repeller, therefore the amplitude of the oscillations depended upon the initial conditions (Figures 1A, 2A; Edelstein-Keshet, 2005).

The oscillations in the original Lotka-Volterra predator prey model were not robust to perturbations, because the system did not contain any negative feedback mechanisms that would have stabilized it. The assumed exponential growth of prey destabilizes the system, as in the absence of predation, prey growth explodes. Predation was assumed to be proportional to the density of prey and thus did not saturate even at very high prey densities, implying the predator could consume prey with zero handling time. These oversimplifications led to the unrealistic result of oscillations that depended on initial conditions. Much of the future work focused on alleviating this deficiency.

### Achieving stable oscillations (limit cycles) in models

The unbounded exponential prey growth was replaced with more realistic functions such as the logistic function or the Gompertz function. The logistic function was first developed by Verhulst (1838) to model growth in human populations, and was subsequently shown by Gause (1934) to be a good fit to the growth of populations of *Saccharomyces cerevisiae*. The differential equation for logistic growth takes the form:


(2) $$\frac{dN}{dt}=\mu N\left(1-\frac{N}{C}\right)$$

Where μ is the specific growth rate and C is the carrying capacity. If the population density is much lower than the carrying capacity (N ≪ C), N / C vanishes, and exponential growth is recovered. With increasing N, growth rate declines linearly, becoming zero when N = C, and negative when N > C. This makes the carrying capacity a stable steady state. The solution of the logistic function, which describes the population dynamics over time, shows a sigmoidal increase of the population from low N, with growth slowing down close to the carrying capacity.

The Gompertz function was first used in population dynamics by Gompertz (1825) to model human mortality figures and has since been adapted for predator–prey models. The differential equation for the Gompertz model takes the form:


(3) $$\frac{dN}{dt}=\mu N\ln\frac{C}{N}$$

An alternative means of constraining prey growth, developed specifically for bacteria by Monod (1949), was to make growth dependent not on population density but on nutrient concentration (which may in turn depend on population density) with specific growth rates to saturate at high nutrient concentrations. In the Monod equation growth rate takes the form:


(4) $$\frac{dN}{dt}=\frac{\mu_{\max}\;S}{S+K}N$$

Where $\mu_{\max}$ is the maximum specific growth rate, S the nutrient or substrate concentration and K the half-saturation constant, that is the concentration of substrate required for the prey’s specific growth rate (μ) to become half $\mu_{\max}$. The Monod equation is a hyperbolic function with increases in substrate concentration having the largest effect when substrate concentrations are low and very little effect at already high substrate concentrations.

For the predator, similar constraints on growth are required for a system to realistically model biology, as no predator can consume prey at a rate proportional to prey density (without saturation caused by positive handling times), as implied by the Lotka-Volterra equations. Alternative forms for the predation rate were developed by Holling whilst studying the predation of sawflies by small mammals (Holling, 1959b), and subsequently tested in the so-called disk experiment, with human volunteers taking the role of predators hunting for disks on a table while being blindfolded (Holling, 1959a). Holling proposed three forms of predation rate or functional responses. In the type I functional response, the predation rate is proportional to prey density up to a threshold, at which point predation rate saturates and does not change with further increase in prey density. This type of functional response is found in filter feeders (Jeschke et al., 2004). The type II functional response takes the same form as the Monod equation (Eq. 4) and results from a handling time when prey is caught, which limits the maximum predation rate at high prey density. The type III functional response has a sigmoidal form and arises from the presence of an alternative prey. Which if any of these functional responses best matches the actual predation rate of a system depends on the nature of predator, prey and their environment, and indeed Holling saw live predation patterns that fitted all three proposed forms (Holling, 1959b).

### Alternative functional responses

The functional responses described above all depend solely on the prey density and do not consider predator numbers. Alternative predator functional responses, which introduce a dependence on predator density, have been proposed. These include a per-capita predation rate (Arditi and Ginzburg, 1989) or the logistic function (Leslie, 1948). The logistic function takes the form of a sigmoidal curve, meaning increases in prey density have greatest effect on predation rate at intermediate prey densities. The logistic function and per-capita predation rates ensure that all trophic levels share the benefits of any increase in nutrients and remove the paradox of enrichment effect, where increasing nutrients for the lowest trophic level destabilizes the system, increasing the amplitude of the oscillations, such that at their nadir the predator and prey populations become very small and are at risk of extinction (Rosenzweig, 1971). Evidence from laboratory experiments and field trials shows that while some live predatory-prey systems (generally those with a homogenous environment, such as a chemostat) are destabilized by an increase in nutrients, others (mostly those with a heterogeneous environment) are not (Huffaker et al., 1963; Arditi and Ginzburg, 1989; Ginzburg and Akçakaya, 1992). Furthermore, whilst mechanisms such as ratio-dependent and per-capita predation rates are sufficient to eliminate the paradox of enrichment, they are not necessary. Other mechanisms, such as predator mortality, which have the greatest effect on overall predation at low prey densities, also give a stabilizing effect (Nisbet et al., 1983).

Another issue to consider when choosing functional responses is the biological control paradox, which deals with whether a prey population can be both very low and at the same time stable (Luck and Fluck, 1990; Arditi and Berryman, 1991). Functional responses based only on prey density are not stable at low densities, whilst those that also include predator density can be. Observations of live predator–prey systems have shown that while some systems are unstable at low prey density others are stable (Turnbull and Chant, 1961; Hagen and Franz, 1973). The conclusion from this is that care should be taken when choosing growth and predation rate functions to select the most appropriate functions as the type of prey and predator growth terms used can significantly impact population dynamics (see Figure 2) and no one function has been found that is best for all scenarios.

Adding saturation to prey growth or predation rates in any of the above forms alters the stability of the system, making oscillations more or less likely, depending on the precise functions chosen. With the original Lotka-Volterra model (Figure 2A), all scenarios result in unstable oscillations around a neutral center (Figure 1B). The addition of constraints on prey and predator growth stabilizes or destabilizes the system allowing a wider range of outcomes. Adding logistic prey growth and a Holling type II functional response to the original Lotka-Volterra model, results in either the elimination of the predator, or oscillations either in the form of a stable limit cycle or linearly unstable region around a saddle node (where sustained oscillations occur around a steady state that is unstable in the simplified, linearized system of equations) (Figures 1B, 2B). Oscillations following the trajectory of a stable limit cycle are classified as stable oscillations according to linear stability analysis, however their period and amplitude may be so extreme as to render the populations vulnerable to extinction.

Oscillations of microbial populations are most easily observed in a chemostat, which in the absence of oscillations can maintain a steady state population level for many weeks. To explore these, we investigated the possible dynamic regimes of four different predator–prey chemostat models. Figure 2C used equations and parameters for protist predation of bacteria from Curds and Bazin (Curds and Bazin, 1977). This system could result in damped oscillations or a steady state, but more usually gave sustained oscillations. The rotifer model by Fussmann et al. (2000) was the only one of these models that was fitted to experimental data. It gave a similar pattern to protist predation, but with a greater likelihood of observing damped oscillations or a stable steady state (Figure 2D). *B. bacteriovorus* predation, based on parameters and equations from Summers and Kreft (2021), had a larger region of damped oscillations, but also a much larger region of linear instability (Figure 2E). The same model parameterised for bacteriophage predation (Figure 2F), gave a very different pattern. Across the parameter ranged tested, there was no region of linear instability and the region in which only the prey survived shrank to a small band.

Many real animal predator–prey systems have also been shown to display oscillations, including snowshoe hares and lynx (Maclulich, 1936; Stenseth et al., 1997), lemmings (Elton, 1943), moths (Bigger, 1973) and protists (Luckinbill, 1973; Blasius et al., 2020) to name but a few. Understanding the nature of these oscillations, and how they are likely to be impacted by interventions, such as nutrient enrichment, or the reduction of predators, has implications for both species conservation and biocontrol of pest species, as well as the use of predators to reduce or eliminate pathogens.

## Mathematical models of predatory bacteria

Only a few studies with mathematical models have involved predatory bacteria, see Table 2 from Summers and Kreft (2021) for an overview of these. The earliest of these, by Varon and Zeigler (1978), used the Lotka-Volterra model (Eq. 1a and 1b) which they could fit to experimental data on the dependence of predation rate on prey density. Using this, Varon predicted a minimum prey density of 7 × 10^5^ cfu ml^−1^ was required to sustain their species of BALO. The data from that study were recently reanalyzed and fitted to a Holling type II functional response by Summers and Kreft, both to provide parameters for their model, which explored the effects of changes in predator or prey characteristics on population dynamics (Summers and Kreft, 2021), and to inform on parameter ranges for model fitting to experimental data (Hobley et al., 2020).

**Table 2:** Previous models of *Bdellovibrio* and other relevant microbial predators, taken from Table S1 in Summers and Kreft (2021) and compared to their principal model 6.

Bdelloplast stage?	Predator Mortality?	Prey Growth	Holling type	Batch or chemostat	Notes	Reference
No	No	Exponential	I	Batch	Lotka-Volterra model	(Varon and Zeigler, 1978)
No	Yes	Monod	I	Chemostat	Delay between predation and birth of predators	(Crowley et al., 1980) Model 1
No	Yes	Monod	I	Chemostat	As Crowley Model 1, but also includes bdellophage (phage that infect *Bdellovibrio*).	(Crowley et al., 1980) Model 2
No	Yes	Monod	II	Chemostat	Protist predation	(Nisbet et al., 1983)
No	Yes	Monod	II	Chemostat		(Wilkinson, 2001) Model 1
Yes	No	Monod	I	Chemostat	As Wilkinson Model 1, but also contains decoys	(Wilkinson, 2001) Model 2
Yes	Yes	Exponential	I	Batch	Contains decoys and nutrient recycling	(Hobley et al., 2006)
Yes	Yes	Monod	I	Batch	Includes effects of serum	(Baker et al., 2017)
Yes	No	Exponential	I	Batch	Gaussian function for bdelloplast maturation	(Said et al., 2019)
Yes	Yes	Monod	II	Chemostat	Family of models with various ingredients examined	(Summers and Kreft, 2021) Model 6

Copyright © 2022 American Society for Microbiology. *Appl. Environ. Microbiol.* 88:e01082-21. doi: 10.1128/AEM.01082-21

More complex models with alternative prey growth terms and predator functional responses have been used in the modelling of protist predation (Canale, 1969; Jost et al., 1973a,b; Curds and Bazin, 1977; Nisbet et al., 1983). The earliest model of bacterial predation to use these was Crowley et al. (1980), who investigated several models with Monod kinetics for prey growth and introduced a delay between prey death and the release of new predators, to account for the lengthy bdelloplast stage. These models, like similar models of protist predation, showed that as well as steady state co-existence, bacterial predation could result in sustained oscillations. Which type of regime was encountered depended on both predator and prey growth characteristics and the ambient conditions (nutrient concentration and flow rate). Both protist and bacterial predation models also showed population dynamics that were destabilized by increased nutrient concentrations, a paradox of enrichment effect (Rosenzweig, 1971), leading to extreme oscillations and a loss of robust permanence.

Whilst the delay-differential equations are interesting from a theoretical point of view, Crowley’s model was not verified by testing against laboratory data. Indeed, there is in general a dearth of experimental studies of bacterial predation in chemostats, which is the chosen setting for the purely theoretical models of *B. bacteriovorus* predation. The theoretical works consider chemostats, because they are energetically open systems in which long-term population dynamics can be studied and dynamic behavior such as oscillations can be observed. To date, the only experimental studies in chemostats were conducted in the late 1970s and early 1980s (Whitby, 1977; Varon, 1979; Dulos and Marchand, 1984; Varon et al., 1984). Whitby (1977) was able to maintain a *B. bacteriovorus* and prey co-culture for up to 3 weeks and observed reproducible patterns of oscillations with a frequency dependent on the dilution rate, as well as a stable steady state at low dilution rates. By contrast, Dulos and Marchand (1984) reported that the oscillations they saw were not reproducible. Varon initially reported a mutant strain of prey arising from the chemostats (Varon, 1979). This mutant grew more slowly, but was resistant to *B. bacteriovorus* predation, resulting in a drop in predator numbers. Theoretically this sort of three-member system could be stable (Cramer and May, 1972), however the experiment was not continued long enough to determine if this particular community was stable. Genetically stable resistance as seen by Varon (1979) is unusual against BALOs, but is frequently seen with other microbial predators and can happen in ecologically relevant timespans (Luria and Delbrück, 1943; Yoshida et al., 2003; Friman et al., 2008; Kasada et al., 2014). In later experiments however Varon and co-workers saw oscillations whose period depended on the substrate concentration (Varon et al., 1984). These few examples illustrate the difficulties in working experimentally with *B. bacteriovorus* in chemostats. Similar issues regarding reproducibility of results have been reported with protist predation, which at certain dilution rates and nutrient levels can show chaotic behavior (Becks et al., 2005). These studies highlight the value of mathematical models to explore situations where it would be difficult or impossible to use an experimental setup.

Subsequent models of bacterial predation have added complexity by either including additional prey or decoy species (Wilkinson, 2001, 2003, 2006; Hobley et al., 2006; Summers and Kreft, 2021), additional predators (Hobley et al., 2020; Summers and Kreft, 2021), a stochastically varying lysis time for the bdelloplast (Said et al., 2019) or more complex environments, such as the effects of predation in human serum that contains a predator killing complement system (Baker et al., 2017; Im et al., 2017b) or spatial effects (Dattner et al., 2017). Wilkinson (2001) noted the failure of predators, such as *B. bacteriovorus*, to eliminate their prey in many microbial communities and sought to test whether this could be due to decoy species such as Gram-positive bacteria. His model predicted that the presence of a decoy benefited the prey, either by eliminating the predator or reducing the amplitude of oscillations in predator and prey densities. Hobley et al. (2006) tested the decoy effect in a batch culture experiment and model and found that the presence of the decoy benefited both the predator and its prey, possibly due to nutrients released when the decoys lysed. They included effects such as cellular crowding and proteases (produced by the decoy cells), which could degrade cellular material into components that could fuel bacterial growth. Whilst this may increase the realism of the system, the addition of multiple new elements to a model can also make it more difficult to pick apart which factors have the greatest effect. Additionally, the use of batch cultures means that long-term trends, such as oscillations, cannot be observed.

Summers and Kreft (2021) also investigated the effects of alternative species, using a mathematical model of a chemostat to explore how additional prey or predator species altered the stability of predator and prey populations. They found that a second prey species could stabilize their system, whilst a second predator species could only be supported in quite specific circumstances. Summers and Kreft also looked at how altering aspects of the predator or prey would affect population dynamics. They studied both the effects of altering a single parameter, such as burst size or predator mortality, as well as more complicated scenarios such as changes to prey cell size, which affected several other parameters. This allowed them to both understand the contribution of a change in a single parameter to the system stability and how biologically relevant combinations of changes would likely impact the prey–predator dynamics. They found a system that was prone to extreme oscillations, likely to result in predator extinction and thus a lack of robust permanence. They also noted that properties that gave maximal predator productivity were close to those resulting in extreme oscillations.

Many predator prey models employ an element of stochasticity (Jeschke et al., 2002; Dobramysl et al., 2018) as a means of removing the simplifying assumption that all organisms in a population respond at the average rate. To our knowledge, however, stochasticity has only been used in one study modelling *B. bacteriovorus* predation, where Said et al. (2019) based the lysis time of bdelloplasts on a Gaussian function and used non-saturating growth terms for both prey and predator. This resulted in bursts in predator numbers at the most likely lysis time, as is often seen in laboratory experiments on *B. bacteriovorus*, however, it also underestimated both the prey and bdelloplast numbers compared to the experimental data.

Baker et al. (2017) and Im et al. (2017b) introduced another level of biological realism by including the effects of human serum on *B. bacteriovorus* predation. Both studies found that the presence of serum caused a delay in predation. Baker et al. (2017) used a complex model, including both predation resistance, changes in predation rate over time and the release of growth supporting nutrients from dead and predated cells. They considered the decrease in predation rate to potentially be due to the presence of both nutrients for the prey and antimicrobial agents in the serum. Alternatively, Im et al. (2017b), who used the Lotka-Volterra model, believed the delay was due to osmolality. Dattner et al. (2017) is to date the only model of *B. bacteriovorus* predation that has looked at spatial effects. They based their mathematical model on their laboratory model employing sand to represent soil, where connectivity between patches depends on water availability (matrix potential). They found that, as has been predicted (Alexander, 1981), the presence of a spatial refugee, more common under dry conditions, can result in prey survival.

While the purely theoretical works used Monod growth kinetics for the prey and a Holling type II functional response for the predators, those papers that map experimental data to their models frequently used the Lotka-Volterra model (Eq. 1) and, with exception of the most recent such paper (Hobley et al., 2020), always had a non-saturating predator response. This distinction is important as the theoretical papers looked at a wide range of “what if” scenarios, including situations that would be difficult to replicate under laboratory conditions, whilst the experimental papers validated models against real world data and have been used to inform on realistic parameter ranges for the theoretical models. If the biological assumptions used by the two types of models differ, then it becomes uncertain whether differences in outcomes are due to differing assumptions or other reasons. As detailed earlier in this review, which type of functional response is most appropriate in a macro-predator situation depends on the nature of the predator. Hobley et al. (2020) showed that this was also the case with microbial predators, as a non-saturating functional response fitted better to bacteriophage predation, whilst a Holling II functional response was a better fit to *B. bacteriovorus*. This difference can be understood by comparing the predators in question. The bacteriophage drifts until it bumps into a prey cell, at which point penetration is rapid (Stent and Wollman, 1952), the lysis time is also short and the burst size large (Hadas et al., 1997). In contrast, *B. bacteriovorus* swims at fast speeds (Lambert et al., 2006), thus increasing its encounter rate with prey, but is slower to enter the prey (Varon and Shilo, 1968) and has a longer lysis time and lower burst size (Seidler and Starr, 1969b). Combined, these physiological factors mean that bacteriophage predation is likely to saturate at a significantly higher prey density than *B. bacteriovorus* predation.

## What can we learn from protist predation?

*B. bacteriovorus* is in many ways a unique predator. It has a long “digestion” phase relative to its hunting phase, predates one prey item in its lifetime and unlike many other predators does not hunt while digesting. It is also smaller than its prey, resulting in a comparatively large burst size. Together these factors mean it demonstrates unusual population dynamics which requires unique modelling terms to fully capture. In many ways the closest comparison is to a bacteriophage, but even here there are noticeable differences, see Table 1. Despite these differences there are biological lessons to be learnt from comparisons with other microbial predators, which have been more fully studied. As an example, it is known that protists predating a single species struggle to eliminate their prey (Danso and Alexander, 1975; Habte and Alexander, 1978b), unless there is a second factor involved, such as the presence of a bacteriostatic antibiotic (Habte and Alexander, 1978a) or an alternative prey species (Mallory et al., 1983). The same phenomenon of residual prey populations is also seen with bacteriophage (Wiggins and Alexander, 1985) and with BALOs (Keya and Alexander, 1974). There are multiple potential reasons for the survival of microbial prey, for fuller details see the review by Alexander (1981). One of the reasons given is the introduction of another trophic level, such that the predators themselves get predated. This was investigated in a purely theoretical sense by Crowley et al. (1980) who introduced a bacteriophage exclusively predating *B. bacteriovorus* into an *E. coli*, *B. bacteriovorus* predator–prey system and found it had little impact on the prey survival. By contrast the introduction of a protist predator (*Tetrahymena pyriformis*) was sufficient to rescue *Klebsiella pneumoniae* prey form extinction when facing competition from other prey bacteria and predation by *B. bacteriovorus* (Johnke et al., 2017). Another suggested reason for prey survival is resistance. Resistance to protist (Meyer and Kassen, 2007) and bacteriophage (Labrie et al., 2010) predation is well known, genetically inherited resistance to BALOs is less common, to our knowledge only two studies (Varon, 1979; Gallet et al., 2009) have reported this to occur. Phenotypic resistance to *B. bacteriovorus* predation (Shemesh and Jurkevitch, 2004) is more common, though poorly understood, however the recent work by Hobley and coworkers combining experimentation and modelling has helped improve this (Hobley et al., 2020).

## What can we learn from optimal foraging theory for *Bdellovibrio bacteriovorus*?

Optimal foraging theory is based on the idea that foraging behavior has evolved to be optimal for the fitness of the consumer. For an overview, see Pyke (1984) and note that this theory, like other theories, has been debated (Pierce and Ollason, 1987; Schmid-Hempel and Stearns, 1987). It is about behavior, which can be genetically determined or learned, not traits. The theory makes six general assumptions:

1. Fitness depends on foraging behavior. This is certainly true for *B. bacteriovorus*, which is a full-time hunter in attack mode without sleeping and then in full-time prey consumption mode.
2. Foraging behavior is at least partly heritable (this could include learnt behavior where the rules are partly heritable). The foraging behavior of *B. bacteriovorus* may be entirely heritable as it only predates once per generation, which should make learning impossible. There may, however, be plasticity in the heritable behavioral repertoire. Prey range is presumably genetically determined as it depends on molecular interactions of the predator with its prey and different strains can have different prey ranges.
3. The relationship between fitness and foraging behavior is known. For *B. bacteriovorus*, there clearly is a relationship between fitness and foraging behavior, and even though it is not fully known it can be studied. When describing behavior in an agent-based model, it is not necessary to know which functional form the relationship takes, as this would be an emergent property of the interactions of individual organisms, and thus an outcome of the model simulations (Colon et al., 2015; Oremland and Laubenbacher, 2015).
4. Evolution of foraging behavior is not prevented by genetic constraints. There is no reason to think that foraging behavior in *B. bacteriovorus* cannot continue to evolve as it clearly has evolved in the past.
5. Evolution of foraging *behavior* is constrained by functional characteristics of the animal that appear ‘fixed’ on the shorter timescale at which behavior evolves. These ‘fixed’ characteristics can be considered to evolve in a broader framework. For *B. bacteriovorus*, it is likely that evolutionary changes of behavior in adaptation to changes in the prey community composition are slower than the changes of the community composition, thus limiting evolutionary adaptability to prey communities.
6. Behavior evolves more rapidly than foraging conditions change such that optimality can be reached, subject to functional constraints. For *B. bacteriovorus*, this is uncertain. It seems likely that many foraging conditions change more rapidly than behavior can evolve since behavior is genetically ‘hard-wired’, but there may be more general foraging conditions in a habitat that change more slowly, such that evolutionary adaptation to these is possible.

In summary, all basic assumptions of optimal foraging theory are at least partially met in *B. bacteriovorus*, so the theory should apply and *B. bacteriovorus* could be used as a model to test the theory. Agent-based models would predict fitness consequences of behaviors, relaxing the need to specify functional relationships or make assumptions about timescales.

Foraging organisms are making four basic choices: what to eat (diet choice), where to forage (patch choice), when to leave a patch and when/how to move within a patch. Optimal *foraging* theory predicts that food items are not eaten if their rank in terms of food gain per time is too low, even if this food item is abundant. Higher overall food abundance should lead to specialization on highly ranked food. For *B. bacteriovorus*, and microbes generally, it would be difficult to choose a patch and to choose to leave a patch, because this requires directed movement on larger scales, in contrast to movement within a patch, which motility and chemotaxis could accomplish. Directed movement over larger distances from one patch to another would require long range ‘vision’ and directed motility that could overcome water or air currents. Currents would transport microbes against their chemotactical ‘wishes’. This leaves diet choice and movement within a patch as the two basic choices for microbial predators. For *B. bacteriovorus*, diet choice beyond sensing a Gram-negative cell surface, may be very limited as encounters with prey are random and the size of the prey cannot be gauged, in contrast to for example a bird that can recognize different seeds, see their size and pick the biggest and tastiest seed. Applying the concepts of optimal foraging theory to microbes has shown promise (Heineman et al., 2008; Fernandez et al., 2019) and we surmise will prove fertile in the future.

## Conclusion and future directions

To date, mathematical modelling of prokaryotic predation has enabled researchers to analyze experimental data, selecting appropriate models and inferring the parameters of predation to better understand the mechanisms and kinetics of the predator prey dynamics, and to ask “what if” questions that would be difficult to investigate in any other manner. Most studies have either used simple models with one or two predator or prey species in a spatially homogenous environment or have fitted their model to one particular experimental setup. For medical applications, mathematical models have helped to look at the challenges likely to be encountered when using predators in a human or animal setting, including decoy species, the presence of human serum and the potential synergies or antagonisms of an additional predator or other antimicrobial agent. Further work should extend on this to include effects of structured and complex spatial environments, the effects of a diversity of other prey (which may act to boost predator numbers) or non-prey species as well as treatment strategies, such as partnering predatory bacteria with antibiotics or other predators or optimizing dosage.

Most prokaryote focused mathematical models have investigated systems based on chemostat conditions (constant inflow of nutrients and removal of organisms at a set dilution rate) as they enable the study of oscillations. These have shed light on the effects of nutrient level and dilution rate on the stability of systems with a small number of microbial species, and have asked “what if” questions based on the growth characteristics of these species. While these models have looked at some basic ecological theories, such as the paradox of enrichment, they have not been applied to investigate whether more complex theories, such as optimal foraging, apply to bacterial predators and if so to what extent. What is principally lacking are the complications of spatial structure, the effects of evolution and selection, and the properties of *B. bacteriovorus* that make it different from bacteriophage (e.g., the effects of motility and chemotaxis, the ability to predate dormant cells – often found deeper in biofilms, and the ability to penetrate a biofilm). By carefully designing both experimental and theoretical work it should be possible to use *B. bacteriovorus* as a model organism to investigate ecological theories, such as optimal foraging, to determine the degree to which properties such as motility and chemotaxis assist *B. bacteriovorus* in non-homogenous spatial environments, and to more fully understand this fascinating predator. To do this however we need to ensure that experimental and theoretical work is iteratively designed as one integrated whole, with experiments testing theories suggested by the modelling, and improvements made to models in light of experimental results.

Our understanding of the genetics and molecular mechanisms as well as the lifecycle and physiology of *B. bacteriovorus* has increased greatly in the 60 years since its discovery. In that time, mathematical models have also developed, but more on the side and not to the same extent. By using modern computing power and more complex techniques, such as individual-based models and Bayesian inference, we can fully exploit the power of mathematical modelling to not only better understand *B. bacteriovorus*, but to optimize applications such as removal of antimicrobial resistant populations from wastewater or farm slurries, treating antimicrobial resistant bacterial infections or more generally to replace antibiotics.

## Author contributions

JKS and J-UK wrote the manuscript. All authors contributed to the article and approved the submitted version.

## Conflict of interest

The authors declare that the research was conducted in the absence of any commercial or financial relationships that could be construed as a potential conflict of interest.

## Publisher’s note

All claims expressed in this article are solely those of the authors and do not necessarily represent those of their affiliated organizations, or those of the publisher, the editors and the reviewers. Any product that may be evaluated in this article, or claim that may be made by its manufacturer, is not guaranteed or endorsed by the publisher.
